# Rapid invisible frequency tagging (RIFT): a promising technique to study neural and cognitive processing using naturalistic paradigms

**DOI:** 10.1093/cercor/bhac160

**Published:** 2022-04-22

**Authors:** Noor Seijdel, Tom R Marshall, Linda Drijvers

**Affiliations:** Max Planck Institute for Psycholinguistics, Nijmegen 6525 XD, The Netherlands; Wellcome Centre for Integrative Neuroimaging, Department of Experimental Psychology, University of Oxford, Oxford OX3 9DU, UK; Max Planck Institute for Psycholinguistics, Nijmegen 6525 XD, The Netherlands

**Keywords:** electroencephalography, frequency tagging, neural oscillations, magnetoencephalography, sensory processing

## Abstract

Frequency tagging has been successfully used to investigate selective stimulus processing in electroencephalography (EEG) or magnetoencephalography (MEG) studies. Recently, new projectors have been developed that allow for frequency tagging at higher frequencies (>60 Hz). This technique, rapid invisible frequency tagging (RIFT), provides two crucial advantages over low-frequency tagging as (i) it leaves low-frequency oscillations unperturbed, and thus open for investigation, and ii) it can render the tagging invisible, resulting in more naturalistic paradigms and a lack of participant awareness. The development of this technique has far-reaching implications as oscillations involved in cognitive processes can be investigated, and potentially manipulated, in a more naturalistic manner.

## Introduction

**Fig. 1:**
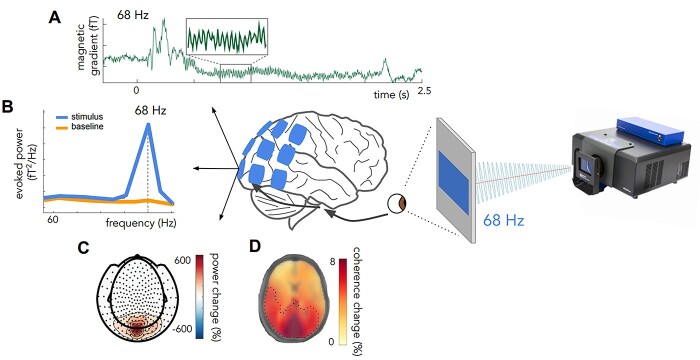
RIFT generates SSVEPs in the brain at the tagging frequencies. A) Event-related fields show responses at the tagged frequency (68 Hz). B) Power at visual sensors shows a peak at the tagged frequency of the visual stimulus C). The power increase at the tagged frequency is strongest over occipital regions. D) Coherence change in percentage when comparing coherence values in a stimulus window to a post-stimulus baseline for 68 Hz. Dotted line indicates region > 5%; A, B, C adapted from Drijvers et al. (2021).

Rhythmic sensory stimulation is a well-established method for studying neural and cognitive processing (Vialatte et al. 2010). For example, presenting a visually flickering stimulus at 6 Hz (or any other low frequency) generates a so-called steady-state visually evoked response (SSVEP) in the brain at that exact frequency (Fig. 1). This frequency tagging method thus allows researchers to track and potentially also probe or manipulate ongoing neural processes with unprecedented granularity and specificity.

Past research has used frequency tagging mostly at lower frequencies (<30 Hz). However, using low-frequency tagging is problematic for 2 reasons. Firstly, low-frequency tagging can be consciously perceived, interfering with task processing. Secondly, this low-frequency tagging potentially entrains or disrupts endogenous neural oscillations in the same range, which have often been linked to cognitive processes including the prediction of upcoming sensory input (Arnal and Giraud 2012; Lewis et al. 2016) and top-down mechanisms that shape the communication between distant regions or networks in the brain (Bastos et al. 2015; Fries 2015; Bonnefond et al. 2017). To overcome these problems, for the past 5 years studies fueled by newly developed projectors with higher refresh rates, tag information at higher frequencies (>60 Hz). This technique is called rapid invisible frequency tagging (RIFT). In this feature article, we provide a brief overview of RIFT applications that could advance our current understanding of oscillations involved in cognitive processes. To showcase the use of RIFT in a broad variety of domains, we highlight recent findings using RIFT and provide an overview of its future promises and challenges.

### RIFT by smooth modulation of sensory input

**Fig. 2:**
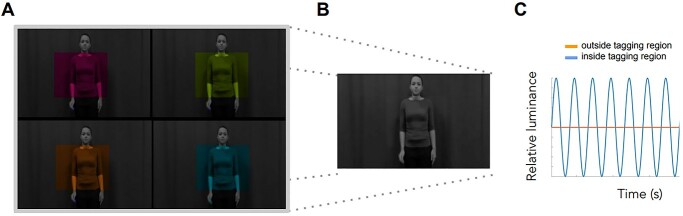
RIFT by the periodic modulation of the luminance of a visual stimulus. A) To achieve smooth sinusoidal modulation at high frequencies, the projector divides each frame received from the graphics card into quadrants, allowing for a 4-fold increase in refresh rate. Color frames in each quadrant can be interpreted by the projector as individual smaller, grayscale frames, which it then projects in rapid succession, leading to a total increase of a factor 12 (4 quadrants * 3 color channels * 120 Hz = 1440 Hz). B) For the participant, a single grayscale stimulus is visible. C) This procedure allows driving the luminance of each pixel with high temporal precision, resulting in smooth sinusoidal modulations without unwanted harmonics.

Frequency tagging is the periodic modulation of a stimulus at a specific frequency, for example by modulating the luminance of a visual stimulus or the amplitude of an auditory stimulus. This frequency-tagged stimulus causes a brain response called the SSVEP (SSVEPs for EEG, or SSVEFs for MEG, or auditory steady state response, in the case of auditory amplitude modulation) with high power at the tagged frequency (Vialatte et al. 2010). When tagging is implemented at frequencies above the human flicker fusion threshold, this is called RIFT. To achieve smooth sinusoidal modulation at these high frequencies, stimuli are presented using special projectors that allow refresh rates of up to 1440 Hz. To achieve this high-frequency mode, the projector (PROPixx DLP light emitting diodes [LED] projector; VPixx Technologies Inc., Saint-Bruno-de-Montarville, Canada) divides each frame received from the graphics card into quadrants, allowing for a 4-fold increase in refresh rate (Fig. 2). Using the 3-color channels (red, blue, green) separately allows for another 3-fold increase, as the color frames in each quadrant can be interpreted by the projector as individual smaller, grayscale frames, which it then projects in rapid succession. The resultant 12-fold increase means that a graphics card with a “base rate” of 120 Hz can achieve a 1440 Hz refresh rate (4 quadrants * 3 color channels * 120 Hz = 1440 Hz). This allows driving the luminance of each pixel with high temporal precision, resulting in smooth sinusoidal modulations without unwanted harmonics. This is different from interpolation techniques in which “impossible frequencies” (frequencies that are not multiples of the refresh rate) are approximated by presenting the stimulus at lower intensities on the frames around the on–off reversal to elicit SSVEPs (Andersen and Müller 2015). With a projector that is capable of presenting stimuli at a refresh rate of 1440 Hz, one could theoretically modulate the luminance of a stimulus at frequencies up to 720 Hz (the Nyquist frequency of the projector). However, so far results have indicated that the neural response to high-frequency flicker drops off sharply around 80–90 Hz.

### The possibilities and advantages of using RIFT

RIFT provides 2 crucial advantages over low-frequency tagging, which we will discuss in more detail below: (i) an increased range of taggable frequencies, leaving low-frequency oscillations unperturbed and open for investigation; (ii) invisibility, resulting in more naturalistic paradigms and a lack of participant awareness.

First, because RIFT tags at higher frequencies (>60 Hz), an important advantage is that the tagging does not interfere with the spontaneous neuronal oscillations in lower frequencies, such as the theta (4–8 Hz), alpha (8–12 Hz) and beta (13–30 Hz) ranges. This renders them unperturbed and therefore allows for the investigation of lower-frequency oscillations during certain cognitive processes (Keitel et al. 2014; Spaak et al. 2014). For example, Zhigalov et al. (2019) used RIFT to investigate neuronal excitability and visual attention in relation to endogenous oscillations in the alpha band. Their results show that RIFT probes brain mechanisms involved in processing of visual stimuli without affecting endogenous oscillations in the alpha range. Similarly, in another study the authors used a novel experimental design to dissociate the effect of attention on the alpha and tagging responses (Zhigalov and Jensen 2020). Here, they found that attentional modulation of alpha power and the frequency tagging response were uncorrelated over trials, suggesting that alpha band oscillations serve a gating role rather than implementing gain control of excitability. Drijvers et al. (2021) used RIFT to investigate (multimodal) integration processes and track lower frequency intermodulation frequencies propagating beyond sensory regions during this process. As a result of the interaction between a visual frequency-tagged signal (gesture; f_visual_ = 68 Hz) and an auditory frequency-tagged signal (speech; f_auditory_ = 61 Hz) they identified an intermodulation frequency at 7 Hz (f_visual_ − f_auditory_) localized to left inferior frontal gyrus (LIFG) and posterior superior temporal sulcus/middle temporal gyrus (pSTS/MTG), areas known to be involved in speech–gesture integration (Drijvers et al. 2021). First studies indicate that one can track responses beyond visual cortex (Drijvers et al. 2021; Marshall et al. 2021). This propagation of the signal cannot just be explained by magnetic field spread, as the tagging signal behaves differently in different regions (Marshall et al. 2021) and can be detected in distal regions, with hemispheric asymmetries in propagation that are in line with task demands (Drijvers et al. 2021, see Fig. 1D). Exactly how far, and to where we can track tagging signals should emerge from future research.

Together, these studies show that RIFT is particularly useful to investigate the role of lower frequency oscillations in cognitive processes, as well as the integration of multiple sensory input streams.

Second, the use of this RIFT ensures participants remain unaware of the stimulus manipulation (and thus often the goal of an experiment), and therefore allows tracking attention to different inputs in a more naturalistic manner. Previous studies using flickering LEDs were already able to tag at frequencies up to 100 Hz (Herrmann 2001). However, using discrete LEDs did not allow for the presentation of complex and naturalistic stimuli. Now, using RIFT, researchers are able to invisibly tag different parts of any type of visual input. The invisibility of the method is influenced by several factors, such as the tagging frequency, the location of the stimulus, the use of static versus moving input or the need for eye movements and the invisibility of the method is indeed yet to be confirmed systematically. Generally, stimuli can be tagged in different regions without obvious distortion of their visual perception. For example, Pan et al. (2021) used RIFT to investigate neural activity associated with lexical parafoveal previewing during natural reading. Compared with previous studies that often involve the manipulation of parafoveal processing or interference with participants' performance, using RIFT allowed them to capture parafoveal processing during more naturalistic reading. Similarly, RIFT may be employed as a window into *hidden* cognitive processes, by illuminating responses that can’t be measured using behavioral methods or conventional imaging techniques. For example, Marshall et al. (2021) used RIFT and MEG to uncover the belief a participant had about an upcoming stimulus before it was presented, i.e. where there was no evoked activity or information in the raw (mean field) signal.

### Future outlooks

Together, these studies already showcase some of the possibilities and advantages of using RIFT in cognitive neuroscience research. Future studies could potentially also use RIFT more directly to influence or manipulate specific oscillatory activity relevant to certain cognitive processes. Being able to *noninvasively* entrain neuronal oscillations opens up a whole new field in which the neuronal oscillations that are thought to be crucial for certain cognitive operations can be manipulated to uncover their causal role in neural processing and cognition. For example, RIFT may be used to manipulate high-frequency oscillations during language comprehension or tasks that require working memory, for example by entrainment of gamma band oscillations. Next to its application in answering more fundamental questions, RIFT could be applied in clinical or therapeutic settings (Iaccarino et al. 2016; Adaikkan and Tsai 2020). For example, changes in gamma oscillations have been observed in several neurological disorders. If RIFT could be used to entrain specific oscillations, this could have important implications for possible interventions, for example targeting oscillatory activity in the gamma range. Finally, many brain–computer interface (BCI) systems nowadays harness the SSVEP measured with EEG. Using RIFT, future studies can boost the bandwidth in BCI systems, resulting in improved performance.

### Limitations

So why isn’t RIFT the answer to everything (yet)? Currently, there are still a few limitations and challenges that need to be considered when setting up an experiment using RIFT. First, given that it is undesirable for tagging frequencies (and their intermodulation frequencies) to interfere with your cognitive process of interest, choosing the appropriate tagging frequencies for your design can be complicated. Secondly, strong responses at the tagging frequencies are typically found in *most* observers, but not *all* observers. Thirdly, the relation between the rapid concatenation of evoked responses (induced oscillatory activity by frequency tagging), neural oscillations entrained by an external perturbation and naturally occurring or endogenous neural oscillations remains to be determined. While their appearance in the M/EEG signal is similar, research has shown that disentangling endogenous versus induced oscillatory activity is not trivial. Evidence for entrainment in general remains equivocal, and the first experiments using RIFT in the gamma range find no evidence for entrainment. (Duecker et al. 2021; Zhigalov et al. 2021). Future work needs to establish whether RIFT entrains neural oscillations (and if so, under which circumstances), and whether those oscillations mechanistically resemble natural oscillations.

## Conclusion

To conclude, the technology enabling RIFT is new, noninvasive, user-friendly and has already produced several interesting and important results. At the same time there is growing interest in tracking dynamic neural representations over fast timescales. RIFT is therefore extremely relevant for cognitive neuroscience as a whole, enabling tracking and manipulation of brain states on rapid timescales and thereby unlocking a next set of fundamental questions.
